# Supplementary material for: Need for self-medication using over-the-counter psychoactive agents: A national survey in Japan
Source: PLoS One. 2021 Jan 25;16(1):e0245866. doi: 10.1371/journal.pone.0245866 (PMC7833153; doi:10.1371/journal.pone.0245866)
Supplement: S1 File — (DOCX) [file pone.0245866.s001.docx]

# S1 File. Items included in the online questionnaire (translated from Japanese to English).

Screening Questions:

1. Do you have any close relationships with individuals who are specialists of psychiatry?

2. Do you have any close relationships with individuals who work at a pharmaceutical company?

3. Have you ever been suffered from psychiatric problems such as anxiety, depression, insomnia, or hallucinations?

Question 1:

Check all items corresponding to conditions that you have previously experienced.

(1) Anxiety (2) Depression (3) Insomnia (4) Hallucinations (5) Others

Question 2:

Check all items corresponding to actions that you have taken to address psychiatric problems.

(1) Consulting a specialist

(2) Consulting a non-specialist

(3) Taking OTC drugs

(4) Taking pharmaceutical drugs prescribed by a doctor

(4) None

Question 3:

Evaluate the effectiveness of each solution you choose above.

(1) Completely ineffective

(2) Relatively ineffective

(3) Fairly effective

(4) Relatively effective

(5) Very effective

Question 4:

To what degree do you believe that OTC drugs are effective for each psychiatric problem (anxiety, depression, insomnia, hallucinations, and others)?

(1) Completely ineffective

(2) Relatively ineffective

(3) Fairly effective

(4) Relatively effective

(5) Very effective

Question 5:

To what degree do you believe that OTC drugs are safe to use for treating each psychiatric problem (anxiety, depression, insomnia, hallucinations, and others)?

(1) Completely unsafe

(2) Relatively unsafe

(3) Fairly safe

(4) Relatively safe

(5) Very safe

Question 6:

Do you agree with each opinion below regarding the advantages of OTC drugs for treating psychiatric problems?

(1) They are affordable and easy to use. [affordable]

(2) They can be used according to one’s health status. [flexible]

(3) They can be used without notifying other people. [private]

(4) OTC drugs do not have serious adverse effects compared with other pharmaceutical drugs. [safe]

(5) I disagree with all of the above.

Question 7:

Do you agree with each opinion below regarding the disadvantages of using OTC drugs to treat psychiatric problems?

(1) It is difficult for individuals to make precise assessments about psychiatric problems. [difficult]

(2) I am afraid of overdosing and/or dependence. [dependence]

(3) I am reluctant to attempt to solve my psychiatric problems using medication. [reluctant]

(4) I am anxious regarding adverse effects that might occur without the supervision of a medical doctor. [risky]

(5) I disagree with all of the above.

Question 8:

Please state your highest level of education.

(1) Junior high school

(2) High school

(3) Job training program

(4) University

(5) No answer

Question 9:

Please state your occupation.

(1) Employed

(2) Government official

(3) Self-employed

(4) Housekeeper

(5) Part-time job

(6) Student

(7) Unemployed

(8) No answer

Question 10:

Please state your work schedule.

(1) Mainly daytime

(2) Mainly nighttime

(3) Shift-work

(4) No regular schedule

(5) No answer
