# Supplementary material for: Need for self-medication using over-the-counter psychoactive agents: A national survey in Japan
Source: PLoS One. 2021 Jan 25;16(1):e0245866. doi: 10.1371/journal.pone.0245866 (PMC7833153; doi:10.1371/journal.pone.0245866)
Supplement: Attachment — Submitted filename: Response to reviewers.docx [file pone.0245866.s002.docx]

Response to the reviewers

Reviewer #1: This is a well-designed study with a novel objective.
However, I would like the authors to address the following points.

*- Major points
I'm wondering OTC drugs available in Japan. The authors may want to show the kinds of OTC drugs and their potential indications for mental conditions.*
**As described in the introduction section, many OTC drugs are available in Japan. But only sleeping pills are categorized as psychiatric OTC drugs. We have added some information about the current availability of OTC drugs in Japan as per the reviewer’s comment.**

*The authors may want to compare their results with previous papers from different countries to discuss the novelty, strengths, consistency, and limitations of this study.*

**Thanks for your advice. When we searched for similar studies conducted previously, we found that very few reports have examined self-medication using OTC drugs in psychiatry. We cited some relevant studies as suggested and included further information about this issue in the discussion section.**

*Please add the limitation of subjective ratings to the discussion section.*
**Thank you for your suggestion. We have added this as a study limitation.**

*I'm wondering why they conclude that Japanese people are lacking in information on OTC drugs for psychiatric problems based on the results of this study.*

**We speculate that people who have experienced psychiatric problems are more likely to encourage the use of OTC drugs because of their experience with such pharmaceuticals and receipt of information from medical practitioners. However, we deleted this sentence from the conclusion since it may be confusing to readers. Thank you for your suggestion.**

- Minor points
*Please describe numbers as mean and SD for continuous variables or numbers and % for categorical variables in the tables.
Please align the number of decimal places.*

**Thank you for your suggestion. We have amended the text in each table.**

*Please ask for a native English check.*

**Thank you for your suggestion. The original manuscript was edited by a proofreading service, but we understand that some confusing phrases may have remained in the text. Before submitting the revised edition, we had the manuscript rechecked by a native English editor.**

Reviewer #2: The paper entitled “Need for self-medication using over-the-counter psychoactive agents: A national survey in Japan” is a well-controlled study and the authors provide new and useful information. Overall I found this study to be interesting and the first study to examine public opinion regarding the use of OTC drugs to treat psychiatric symptoms. However, there are some issues that the authors should address.

*1. The authors described that “Further research and education are required to encourage self-medication of psychiatric symptoms using OTC drugs” in conclusion. The authors should give an example and explain in detail for the pharmacists especially. Please also discuss the relationship between doctors and pharmacists.*

**Thank you for your suggestion. We added some information about the importance of education regarding OTC drugs and factors related to the promotion of OTC drugs, including the importance of collaborations between physicians and pharmacists, in the discussion section. However, few previous studies have examined this issue in Japan, so we were unable to give specific examples.**

*2. In Overseas, many health foods and/or supplements are widespread, and then widely used. In Japan, it seems that their amount used and kinds are less than those in oversea. The author should discuss not only OTC but also health foods and supplements as self-medication. How much they are used as self-medication for psychoactive agents. There are some health foods and supplements used for symptoms.*

**Thank you for your suggestion. We are aware that OTC supplements may have an important role in the issue of self-medication. Unfortunately, we did not gather data regarding supplements in the present study. To our knowledge, few studies have examined this issue in Japan. In the last part of the discussion section, we added some information regarding relevant ideas for further studies.**
